# An FPGA-Based Silicon Neuronal Network with Selectable Excitability Silicon Neurons

**DOI:** 10.3389/fnins.2012.00183

**Published:** 2012-12-24

**Authors:** Jing Li, Yuichi Katori, Takashi Kohno

**Affiliations:** ^1^Graduate School of Engineering, The University of TokyoTokyo, Japan; ^2^Institute of Industrial Science, The University of TokyoTokyo, Japan; ^3^FIRST, Aihara Innovative Mathematical Modelling Project, Japan Science and Technology AgencyTokyo, Japan

**Keywords:** silicon neuron, silicon synapse, digital silicon neuronal network, FPGA, associative memory, synchrony

## Abstract

This paper presents a digital silicon neuronal network which simulates the nerve system in creatures and has the ability to execute intelligent tasks, such as associative memory. Two essential elements, the mathematical-structure-based digital spiking silicon neuron (DSSN) and the transmitter release based silicon synapse, allow us to tune the excitability of silicon neurons and are computationally efficient for hardware implementation. We adopt mixed pipeline and parallel structure and shift operations to design a sufficient large and complex network without excessive hardware resource cost. The network with 256 full-connected neurons is built on a Digilent Atlys board equipped with a Xilinx Spartan-6 LX45 FPGA. Besides, a memory control block and USB control block are designed to accomplish the task of data communication between the network and the host PC. This paper also describes the mechanism of associative memory performed in the silicon neuronal network. The network is capable of retrieving stored patterns if the inputs contain enough information of them. The retrieving probability increases with the similarity between the input and the stored pattern increasing. Synchronization of neurons is observed when the successful stored pattern retrieval occurs.

## 1 Introduction

The nervous system transmits signals by cooperation between neurons and synapses. The neuron generates an overshoot of its membrane potential (spike) when stimulated by a sufficient large current. The waveform is distributed to the synapse and causes neuronal transmitters to be released. The information processing in the nerve system is autonomous, flexible, and robust against various signal distortions. The silicon neuronal network is designed to reproduce activities of the nerve system in real-time. Compared to the current computers, the silicon neuronal network is based on the parallel and distributed processing mechanism rather than the serial centralized framework. This distinctive computational style is expected to allow real-time and large-scale processing of advanced task similar to that in the nerve system (Mallik et al., 2005; Mitra et al., 2009). Besides, the hybrid network constructed with the silicon and the biological neurons is investigated to learn complex behaviors in neurons (Le Masson et al., 2002). A silicon half-center oscillator composed of silicon neurons is proposed for application as an embedded biomedical device and a motion controller (Simoni and DeWeerth, 2007).

The ionic-conductance-based model of a neuronal cell describes its dynamics of ions and ionic channels as exactly as possible. Though equations of this type of models are generally complex, it can reproduce neuronal dynamics considerably precisely. Success of the first one, the Hodgkin-Huxley model (Hodgkin and Huxley, 1952), gave rise to various neuron models of this type. Silicon neurons that implement this type of models can reproduce the complex neuronal behaviors, including bursting, tonic firing, and so on (Mahowald and Douglas, 1991; Simoni et al., 2004; Yu and Cauwenberghs, 2010). The integrate-and-fire (IF) model aims to describe the spike generation in neurons with simple equations without taking ionic dynamics into account (Lapicque, 1907). Later, a leakage term was incorporated to describe attracting nature of the resting state, which formulated the leaky IF (LIF) model. It is an efficient and compact model but with the tradeoff of dynamics. Some of neuromorphic chips that implement neuronal networks with LIF neurons are low power for real-time simulation and conveniently applicable to various applications, optimization, recognition, and memory (Chicca et al., 2007; Chakrabartty, 2010; Arthur et al., 2012). Several efforts to reduce the limitation in the dynamics of the LIF model resulted to expanded LIF models including generalized (Jolivet et al., 2004), exponential (Brette and Gerstner, 2005), and quadratic (Izhikevich, 2006) IF models. They were implemented to realize simple silicon neurons that can produce variety of neuronal activities such as spike-frequency adaptation and autonomous bursting (Rubin et al., 2004; Indiveri et al., 2010; van Schaik et al., 2010). However, the limited structure in the LIF model prevents realizing the property of Class II neurons in the Hodgkin’s classification. A quadratic IF model proposed by Izhikevich (IZH) successfully simulates a wide variety of neuronal activities by combination of a two-variable differential equation and reset of the state variables. Whereas most of the above silicon neurons are realized by the analog electronic circuit technology, there are several digital circuit implementations of the LIF (Indiveri et al., 2011) and the IZH (Cassidy and Andreou, 2008; Thomas and Luk, 2009) models. One of them succeeded to realize a large-scale network with 1024 neurons on a single FPGA chip. In these implementations of the IZH model, the equations are solved using the floating point operation. One of the important points in realizing digital silicon neurons that can simulate various neuronal activities with compact and simple circuits, is to select a neuron model with such capability and find a suitable circuit for its implementation. The IZH model is considerably a good selection because its non-linearity is only second order and it can be implemented with fewer multipliers than other models with the similar capability. This model, however, is not fully capable of realizing the graded responses to the stimulus of Class II neurons. This is because the IZH model approximate the spike process by reset of the state variables, which leads to very similar spikes in response to various stimulus. For example, the maximum membrane potential values in spikes are uniform (30 mV). Another neuron model named a mathematical-structure-based Digital Spiking Silicon Neuron (DSSN) model was proposed (Kohno and Aihara, 2007). This model was designed to simulate several classes of neurons by simple digital arithmetic circuits. It was demonstrated that complex behaviors similar to those in a brain area can be reproduced by an implementation by fixed point operation circuits, which is expected to reduce the hardware resource requirement in circuit implementation. Because this model does not approximate the spike process by the reset of the state variables, it can realize more effectively the graded responses of Class II neurons than the IZH model. Because the transmitter release at the chemical synapses is controlled by the membrane potential at the axon terminal, the graded response property of the neuronal cells is reflected to the amount of synaptic transmitter release, which is modeled in our silicon synapse as illustrated in Figure 3. With the DSSN model in Class II mode, the information of input signal is more directly reflected in the transmitter release than the other 2 models. With the neuronal models with resetting of the state variables including the IZH model (Figure 3C), this property is almost ignored although there is a possibility that it plays some roles in the information processing in the nerve system.

We implemented a network of the DSSNs and silicon synapses on a FPGA device. We have developed a silicon synapse model based on the kinetic ones in (Destexhe et al., 1998) that describes the transmitter release in the presynapse and information of duration of a spike. To demonstrate that our implementation is operating appropriately, we executed an auto-associative memory task, retrieving a memorized pattern by its fragments, which has been widely investigated theoretically (Hopfield, 1982; Knoblauch and Palm, 2001; Sudo et al., 2009). Behaviors of the associative memory in our network are evaluated by an overlap index (Domany and Orland, 1987; Aoyagi, 1995). Synchrony of neurons is also investigated by another index, the phase synchronization index (PSI; Rosenblum et al., 2001). Similar retrieving ability is also shown in a network (Arthur et al., 2012) which is composed by the LIF model based silicon neurons. The LIF model can realize only Class I neurons whereas the DSSN model in our network can simulate both Class I and II neurons by selecting appropriate parameters. In this paper, we report the comparison between the performance of auto-associative tasks in the networks composed of Class I and II neurons.

This paper is organized as follows: In the second section, the model of our silicon neuron and its bifurcation structure are introduced firstly. Then the model of our silicon synapse is presented. We explain the architecture of the implementation of our silicon neuronal network thirdly, including its pipeline structure that improves the efficiency of circuit area occupation. Fourthly, we discuss our FPGA implementation and blocks of bidirectional data transfer with a PC. The experiment results and their analysis are followed as the third section. The conclusion section follows where summary, discussion, and views of our future work are presented.

## 2 Materials and Method

### 2.1 Silicon neuron model

**Figure 1:**
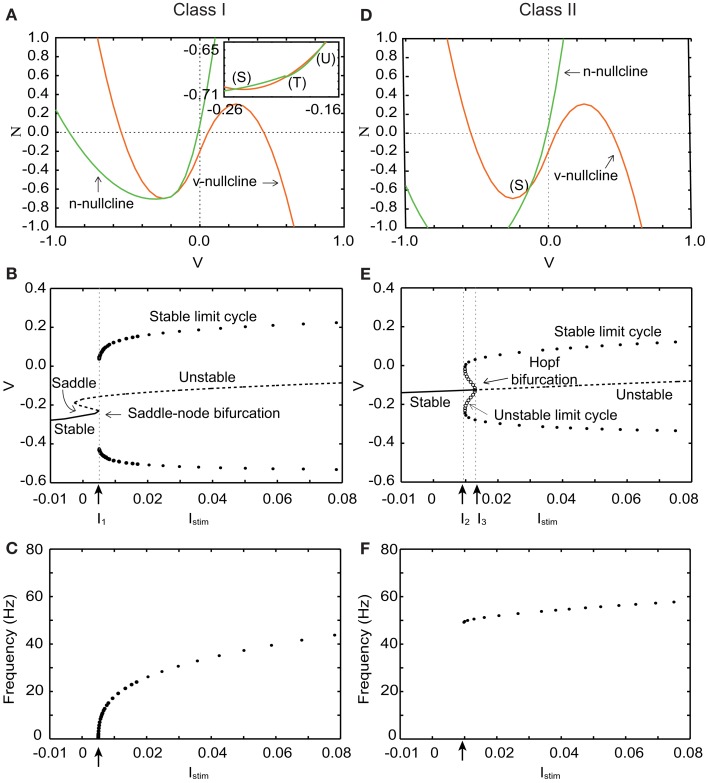
**Phase planes, bifurcation diagrams, and firing frequency of our silicon neuron model in its Class I and Class II modes**. **(A)** Phase plane in the Class I mode. *I*_stim_ = 0. **(B)** Bifurcation diagram in the Class I mode, where the bifurcation parameter is *I*_stim_. *I*_1_ = 0.005. **(C)** Frequency of repetitive firing in the Class I mode. **(D)** Phase plane in the Class II mode. *I*_stim_ = 0. **(E)** Bifurcation diagram in the Class II mode. *I*_2_ = 0.009, *I*_3_ = 0.013. **(F)** Frequency of repetitive firing in the Class II mode.

We adopted the DSSN model (Kohno and Aihara, 2007) for the silicon neurons in our silicon neuronal network system. It is a qualitative model designed from the viewpoint of the non-linear dynamics, which includes sufficient dynamical structure to realize the dynamics of Class I and II neurons in the Hodgkin’s classification (Hodgkin, 1948). It is optimized for implementation by digital arithmetic circuits and defined by two-variable differential equations shown as follows:

$$\begin{array}{llll} \frac{dv}{dt} & =\frac{\varphi }{\tau }\left(f\left(v\right)-n+I_{0}+I_{\text{stim}}\right), & & \text{(1)}\\ \frac{dn}{dt} & =\frac{1}{\tau }\left(g\left(v\right)-n\right), & & \text{(2)}\\ f\left(v\right) & =\left\{\;\begin{array}{cc} a_{n}{\left(v+b_{n}\right)}^{2}-c_{n} & \text{when}\;v<0,\\ -a_{p}{\left(v-b_{p}\right)}^{2}+c_{p} & \text{when}\;v\geq 0, \end{array}\right. & & \text{(3)}\\ g\left(v\right) & =\left\{\;\begin{array}{cc} k_{n}{\left(v-p_{n}\right)}^{2}+q_{n} & \text{when}\;v<r,\\ k_{p}{\left(v-p_{p}\right)}^{2}+q_{p} & \text{when}\;v\geq r, \end{array}\right. & & \text{(4)} \end{array}$$

where *v* and *n* denote the membrane potential and a slow variable that abstractly represents the activity of ionic channels, respectively. Parameter *I*_0_ is a bias constant. In Eq. (1), *I*_stim_ is the weighted sum of the postsynaptic inputs from silicon synapses. Parameters Φ and τ are time constants. Parameters *r*, *a_x_*, *b_x_*, *c_x_*, *k_x_*, *p_x_*, *q_x_* for *x* = *n* and *p*, are constants that control the nullclines of the variables. All the variables and constants in Eq. (1) are abstracted and do not have a physical unit. By selecting appropriate values for these parameters, both Class I and II neurons can be realized with parameter set in Tables 1 and 2. Their phase planes, bifurcation diagrams, and firing frequency are shown in Figure 1.

**Table 1:** **Parameters for Class I mode**.

Par.	Value	Par.	Value
*a_n_*	8.0	*a_p_*	8.0
*b_n_*	0.25	*b_p_*	0.25
*c_n_*	0.5	*c_p_*	0.5
*k_n_*	2.0	*k_p_*	16.0
*p_n_*	−2^−2^ − 2^−4^	*p_p_*	2^−5^ − 2^−2^
*q_n_*	−0.705795601	*q_p_*	−0.6875
Φ	1.0	τ	0.003
*r*	−0.205357142	*I*_0_	−0.205

**Table 2:** **Parameters for Class II mode**.

Par.	Value	Par.	Value
*a_n_*	8.0	*a_p_*	8.0
*b_n_*	0.25	*b_p_*	0.25
*c_n_*	0.5	*c_p_*	0.5
*k_n_*	4.0	*k_p_*	16.0
*p_p_*	−2^−1^ − 2^−4^	*p_p_*	2^−5^ − 2^−2^
*q_n_*	−1.317708517	*q_p_*	−0.6875
Φ	0.5	τ	0.003
*r*	−0.104166	*I*_0_	−0.23

Figure 1A shows the *v* − *n* phase plane of our silicon neuron model in its Class I mode when *I*_stim_ = 0. There are three crossing points between the *v*- and the *n*-nullclines. They are a stable equilibrium (S), an unstable saddle point (T), and an unstable equilibrium (U) from left to right, respectively. Point (S) that corresponds to the resting state attracts the state point located near to it, while (U) repels it. And (T) is known to involve crucially to the mechanism of the threshold phenomena of the spike generation. Points (S) and (T) approach each other if *I*_stim_ is increased. They coalesce and disappear when *I*_stim_ reaches *I*_1_, which is called a saddle-node bifurcation. Point (U) is the only equilibrium point when *I*_stim_ gets larger. Figure 1B shows a bifurcation diagram of our model in the Class I mode, where *I*_stim_ is the bifurcation parameter. It allows overviewing the relationship between dynamics of *v* and value of *I*_stim_. While *I*_stim_ < *I*_1_, *v* converges to (S), which is the only stable state. The limit cycle is generated when *I*_stim_ = *I*_1_ whose maximum and minimum values of *v* are plotted in this figure. If *I*_stim_ > *I*_1_, *v* oscillates along this limit cycle.

Figure 1C shows firing frequency in the Class I mode. The repetitive firing starts with an arbitrarily zero frequency at the bifurcation point because the moving speed of the state point near the saddle-node bifurcation point is slow and decreases to zero when *I*_stim_ is decreased to *I*_1_. And it increases monotonically if *I*_stim_ is increased, which is the property of the Class I neuron.

Figure 1D shows the *v − n* phase plane in the Class II mode when *I*_stim_ = 0. The *v*- and the *n*-nullclines cross each other at a point, stable equilibrium (S). It changes from stable to unstable when *I*_stim_ = *I*_3_ via the Hopf bifurcation. The number of intersections is always one, which is independent of *I*_stim_. In a bifurcation diagram of Figure 1E, a limit cycle appears if *I*_stim_ increases and reaches *I*_2_. When *I*_stim_ is located between *I*_2_ and *I*_3_, neurons have two stable states, a resting state and a stable limit cycle that corresponds to periodical firing. Our neuron fires if *I*_stim_ increases above *I*_3_ no matter where the initial state is. In this case, repetitive firing starts with a non-zero frequency because there is no mechanism that reduces the speed of the state point (see Figure 1F), which is the property of the Class II neuron.

### 2.2 Silicon synapse model

As described in the introduction, our silicon synapse is based on the kinetic synapse model (Destexhe et al., 1998). The synaptic process in response to a single pulse input in our silicon neuron is described by the following equation.

(5) $$\frac{d{Ĩ}_{s}}{dt}=\tilde{\alpha }\left[T\right]\left(1-{Ĩ}_{s}\right)-\beta {Ĩ}_{s},$$

where, ${\tilde{I}}_{\text{S}}$ and [*T*] represent the postsynaptic current and the amount of the released transmitter per impinging spike, respectively. Parameters $\tilde{\alpha }$ and β are the forward and the backward rate constants which represent the rate of the receptors transitioning from the closed state to the open state and its opposite, respectively. We assume that [*T*] has rectangular pulse waveform whose maximum value is 1 and minimum value is 0, in similar way as in (Destexhe et al., 1998). The value of [*T*] is determined by the membrane potential of the presynaptic neuron; the pulses of [*T*] starts when the membrane potential crosses over the threshold voltage (0 in this article) and ends when it crosses down the threshold. For simplification, we defined a new variable $I_{s}=\frac{\tilde{\alpha }+\beta }{\tilde{\alpha }}{Ĩ}_{s}.$ Then we get the following equation.

(6) $$\frac{dI_{s}}{dt}=\left\{\;\begin{array}{cc} \left(\tilde{\alpha }+\beta \right)\left(1-I_{s}\right) & \text{when}\;\left[T\right]=1,\\ -\beta I_{s} & \text{when}\;\left[T\right]=0, \end{array}\right.$$

It can be written as follows, if we define a new constant $\alpha =\tilde{\alpha }+\beta .$

(7) $$\frac{dI_{s}}{dt}=\left\{\;\begin{array}{cc} \alpha \left(1-I_{s}\right) & \text{when}\;\left[T\right]=1,\\ -\beta I_{s} & \text{when}\;\left[T\right]=0, \end{array}\right.$$

The effect of this scaling factor $\frac{\tilde{\alpha }+\beta }{\tilde{\alpha }}$ can be canceled by another coefficient *c* in Eq. (8).

**Figure 2:**
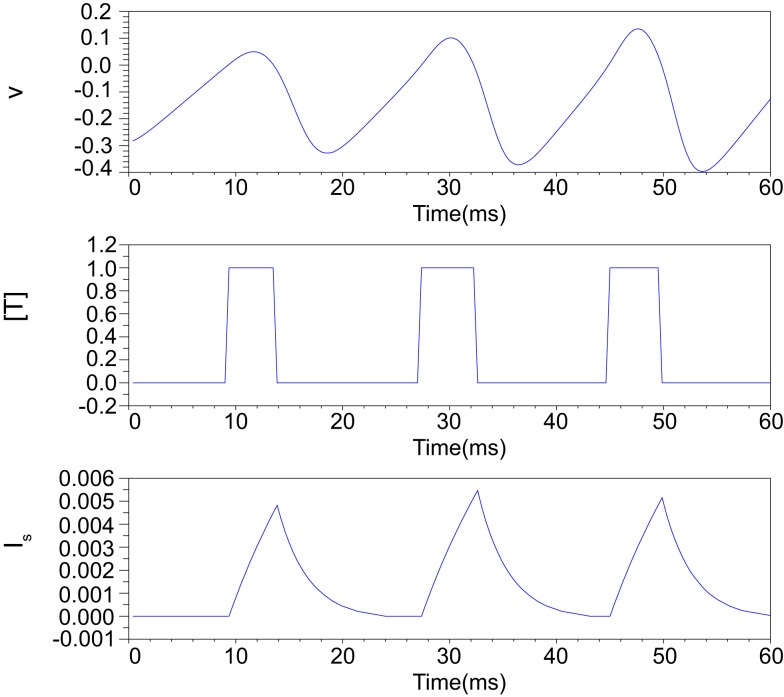
**Numerical simulation of postsynaptic current generation of the Class II neuron**. *I*_stim_ = 0.04 when 0 < *t* ≤ 18.75 ms; *I*_stim_ = 0.06 when 18.75 < *t* ≤ 37.5 ms; *I*_stim_ = 0.08 when *t* > 37.5 ms, α = 83.3, β = 333.3.

Figure 2 illustrates an example of this simplified synaptic activity when the presynaptic neuron is in the Class II mode and α = 83.3 and β = 333.3. The stimulus input *I*_stim_ applied here is 0.04 for the first 18.75 ms and increases to 0.06 until 37.5 ms and finally equals to 0.08. The exponential growth and decay of the postsynaptic input depends on the time duration of the transmitter release.

**Figure 3:**
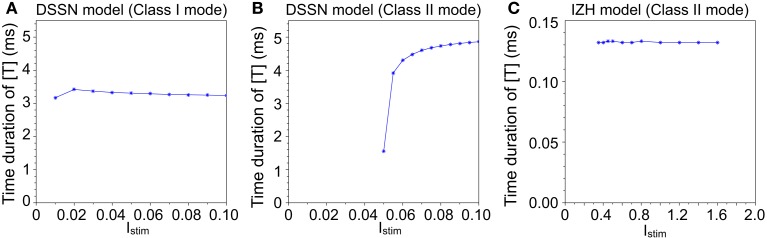
**The time duration of the transmitter release in our silicon synapse model connected to the DSSN model in (A) Class I and (B) Class II modes and (C) the IZH model**. Each neuronal model is in the oscillatory state in response to the sustained stimulus *I*_stim_.

Figure 3 illustrates the time duration of the transmitter release in our silicon synapse model connected to the DSSN and the IZH models. The neuron models are at oscillatory state in response to sustained stimulus current *I*_stim_. It is apparent that with the DSSN model in the Class II mode (Figure 3B), our silicon synapse model can transmit more detailed information of the *I*_stim_ than with the other mode (Figure 3A) and the IZH model (Figure 3C).

The weighted sum of the postsynaptic input *I*_stim_ in Eq. (1) is calculated by Eq. (8).

(8) $$I_{\text{stim}}^{i}=c\overset{N}{\underset{j=1}{\sum }}W_{ij}I_{s}^{j}$$

where, parameters *i* and *j* are the indices of the neurons, $I_{\text{stim}}^{i}$ is the stimulus input of neuron *i* and *N* is the number of neurons. The parameter *c* is a coefficient used to scale *I*_stim_ into an appropriate range and ensure that neurons fire regularly. It equals to 0.060546875 for Class I mode and 0.03125 for Class II mode in this paper. Weight *W_ij_* indicates the strength of the connection from neuron *j* to neuron *i*. The larger absolute value of the weight *W_ij_* means the stronger effect from neuron *j* to *i*. A neuron excites (inhibits) its postsynaptic neuron if *W_ij_* is positive (negative).

### 2.3 Architecture of silicon neuronal network

**Figure 4:**
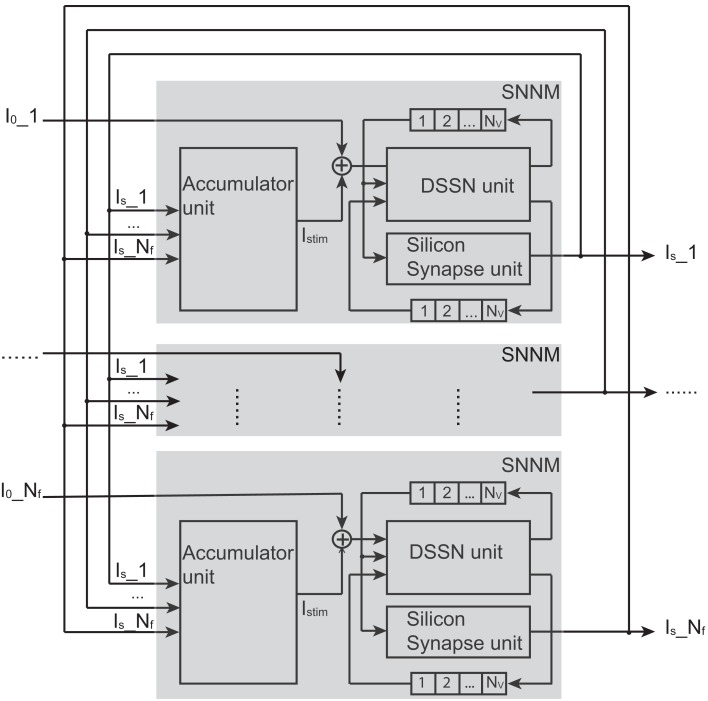
**Structure of our silicon neuronal network**. The network includes multiple (*N_f_*) silicon neuronal network modules (SNNMs). An SNNM executes calculation for multiple *N_v_* silicon neurons and synapses sequentially. The SNNM is composed of three units: a DSSN, a silicon synapse, and an accumulator units. The DSSN unit calculates the membrane potential *v* which is received by the silicon synapse unit to generate the postsynaptic input *I_s_*. An accumulator unit in an SNNM calculates the weighted sum of *I_s_* and added with the bias constant *I*_0_ gives stimulus current to the DSSN unit in the same SNNM.

We designed a Hopfield-type silicon neuronal network in which neurons connect to all the other neurons and whose block diagram is shown in Figure 4.

It is composed of multiple (*N_f_*) silicon neuronal network modules (SNNMs). An SNNM executes calculation for multiple *N_v_* silicon neurons and synapses sequentially. The SNNM is composed of three units: a DSSN, a silicon synapse, and an accumulator units. The DSSN unit calculates the membrane potential *v* which is received by the silicon synapse unit to generate the postsynaptic input *I_s_*. An accumulator unit in an SNNM calculates the weighted sum of *I_s_*, which is added with the bias constant *I*_0_ to give stimulus current to the DSSN unit in the same SNNM. Equations (9–11) show the function of the DSSN unit and the silicon synapse unit configured with the Class I neuronal parameters (see in Tables 1 and 2 for values of Class I and Class II parameters). Where, Δ*t* equals 0.000375 in these equations.

$$\begin{array}{llll} & v\left(t+\Delta t\right) & & \\ & =\;\left\{\begin{array}{c} v\left(t\right)+\frac{\Delta t\varphi }{\tau }\left(8v^{2}\left(t\right)+4v\left(t\right)-n\left(t\right)+I_{0}+I_{\text{stim}}\right)\;\\ \;\text{when}\;v<0,\;\\ v\left(t\right)+\frac{\Delta t\varphi }{\tau }\left(-8v^{2}\left(t\right)+4v\left(t\right)-n\left(t\right)+I_{0}+I_{\text{stim}}\right)\;\\ \;\text{when}\;v\geq 0,\;\\ \; \end{array}\right. & & \text{(9)}\\ & n\left(t+\Delta t\right) & & \\ & =\;\left\{\begin{array}{c} n\left(t\right)+\frac{\Delta t}{\tau }\left(2v^{2}\left(t\right)+v\left(t\right)+\frac{1}{4}v\left(t\right)-\frac{16728}{2^{15}}-n\left(t\right)\right)\;\\ \;\text{when}\;v<r,\;\\ n\left(t\right)+\frac{\Delta t}{\tau }\left(16v^{2}\left(t\right)+8v\left(t\right)-v\left(t\right)+\frac{2560}{2^{15}}-n\left(t\right)\right)\;\\ \;\text{when}\;v\geq r,\;\\ \; \end{array}\right. & & \text{(10)} \end{array}$$

$$\begin{array}{rcll} I_{s}\left(t+\Delta t\right)=\;\left\{\begin{array}{cc} I_{s}\left(t\right)+\Delta t\alpha \left(1-I_{s}\left(t\right)\right)\; & \text{when}\;\left[T\right]=1,\\ I_{s}\left(t\right)-\Delta t\beta I_{s}\left(t\right)\; & \text{when}\;\left[T\right]=0,\\ \; \end{array}\right. & & & \text{(11)} \end{array}$$

**Figure 5:**
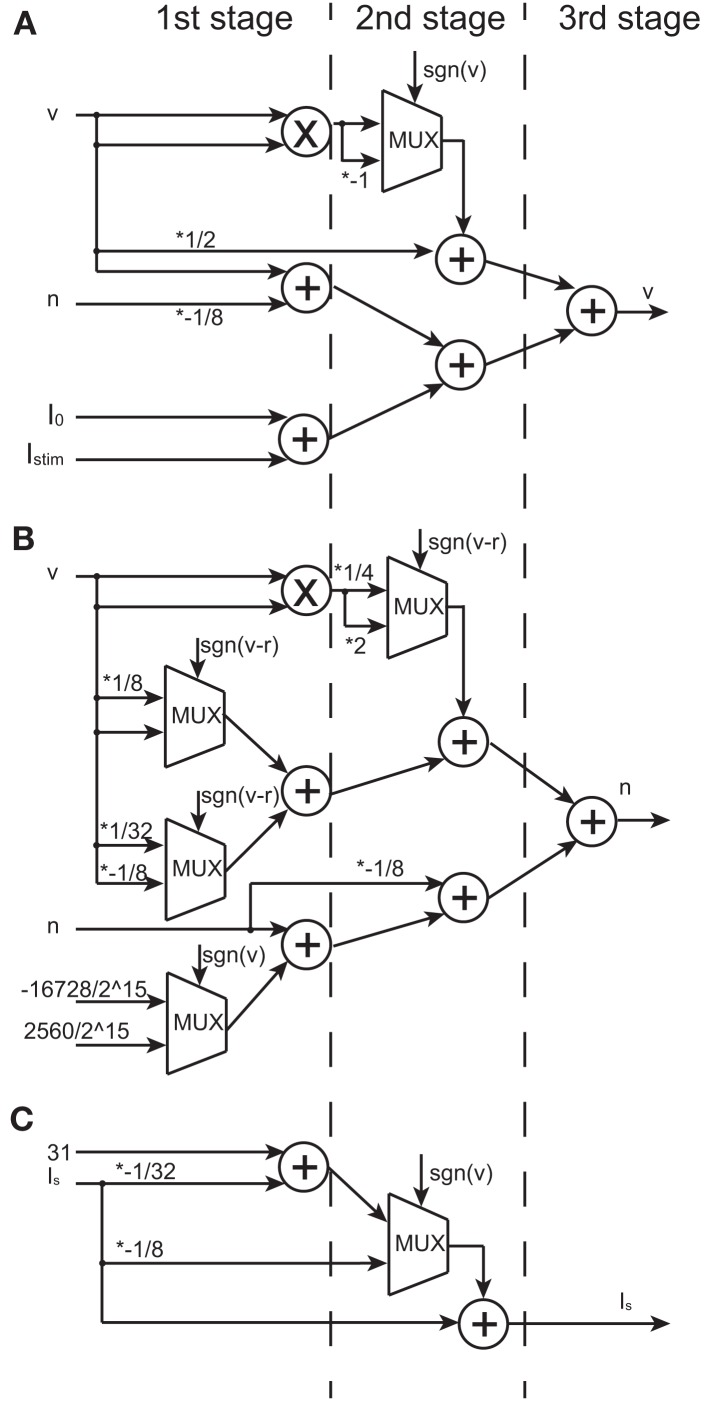
**Block diagrams of the *v*-, *n*-, and *I_s_*-circuits**. Symbols ×, +, and *MUX* mean a multiplier, an adder, and a multiplexer, respectively. Selection signal to the multiplexer sgn(*v*) is the sign bit of *v* and the same to *sgn*(*v*–*r*). Values marked with * represent the multiplication that is realized by a right or a left shift operation. Multiplication by a negative value is realized by multiplication by its absolute and then bit inversion and increment. **(A)** The block diagram of the *v*-circuit, which costs 1 multiplier, 5 adders, and 1 multiplexer. **(B)** The block diagram of the *n*-circuit, which costs 1 multiplier, 5 adders, and 4 multiplexers. The logic units in **(A,B)** are compiled into the 3-stage pipeline structure. **(C)** The block diagram of the *I_s_*-circuit, which costs 2 adders and 1 multiplexer. This unit has the 2-stage pipeline structure. A multiplier is shared in **(A,B)** because their inputs are the same.

The multiplication operation can be replaced by a shift operation if the multiplier is a power of two, by which the required hardware resource is reduced. In the DSSN model, the value of the parameters was selected to realize the bifurcation structure but not the detailed waveform of spikes. We could find appropriate values for the coefficients in our model equations in powers of 2 and sums of 2 power-of-2 numbers. Other parameters like *q_n_* and *q_p_* are not powers of 2 because they are not coefficients and are not involved in the multiplication. Here, we represent *v* and *n* using 18-bit signed fixed point with 15-bit fractions. We chose 18-bit based on the size of the multipliers in commonly used FPGA devices in these days. We confirmed that our silicon neuronal network does not change its dynamic behaviors when bit precision is increased. Figures 5A,B show block diagrams of the circuits that calculate the right-hand side of Eqs (9) and (10), the *v*- and the *n*-circuits, respectively. Symbols ×, +, and *MUX* in the figure represent a multiplier, an adder, and a multiplexer, respectively. A multiplexer selects one of input signals with the control signal and forward the selected input to the output port. A multiplier is shared for all the multiplicative operations because they share the same input *v*. Therefore, a DSSN costs 1 multiplier, 10 adders, and 5 multiplexers. These logic units are classified with 3 stages which run in sequence and cost 3 clocks. The postsynaptic input *I_s_* is calculated by a circuit whose block diagram is illustrated in Figure 5C, which is composed of 2 adders, 1 multiplexer without the multiplier, and they run within 2 clocks.

**Figure 6:**
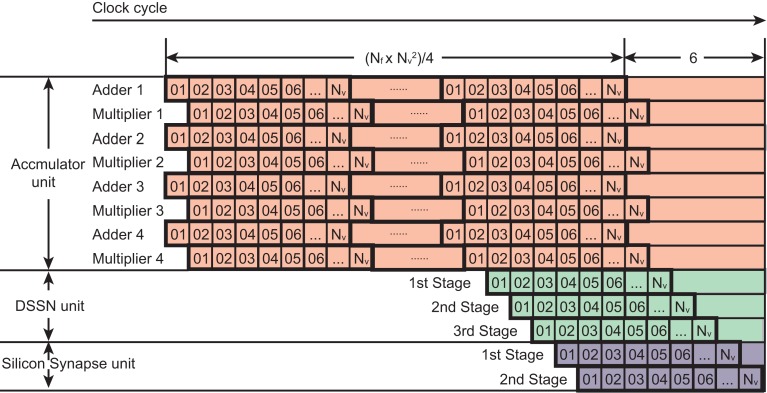
**Clock cycles for an update step of our network**. The horizontal axis is the clock cycle. Each square labeled with a number corresponds to one clock. Each row represents a logic unit utilized in a SNNM. The logic units, 4 adders and 4 multipliers which belong to the accumulator unit cost $\frac{N_{f}\times N_{v}^{2}}{4}+1$ clocks because $\left(N_{f}\times N_{v}-1\right)\times N_{v}$ addition and $N_{f}\times N_{v}^{2}$ multiplication are required in an accumulator unit. The first stage in the DSSN unit runs at the $\left(\frac{N_{f}\times N_{v}^{2}}{4}-N_{v}+3\right)th$ clock, just after the calculation of *I*_stim_ of the first neuron. The second and the third stages run with 1 clock and 2 clocks delay after it. In the silicon synapse unit, the first stage starts running after the membrane potential *v* of the first neuron is obtained in the DSSN unit. So all of the logic units finish their calculation at the $\left(\frac{N_{f}\times N_{v}^{2}}{4}+6\right)th$ clock.

In an accumulator unit, each update step needs (*N_f_* × *N_v_* − 1) × *N_v_* addition and $N_{f}\times N_{v}^{2}$ multiplication operations according to the Eq. (8) and all these operations can be done within $N_{f}\times N_{v}^{2}+1$ clocks because an adder circuit integrates the result of the multipliers that are operated in parallel. We used parallel structures to execute this large number of operations and reduce the number of clocks in a step. For example, if we use 4 multipliers and adders in parallel, the clock number is reduced to 1/4 except for the last clock for an addition. The time cost of one update step for the DSSN unit and the silicon synapse unit is *N_v_* + 4 clocks. Figure 6 shows the clock cycles for one update step of the network. These three units execute the calculation in their pipelined structure. The accumulator unit calculates *I*_stim_ from the 1*st* clock until the $\left(\frac{N_{f}\times N_{v}^{2}}{4}+1\right)th$ clock. The DSSN unit starts running at the $\left(\frac{N_{f}\times N_{v}^{2}}{4}-N_{v}+3\right)th$ clock and total costs *N_v_* + 2 clocks because it contains 3 stages as shown in Figure 5. While the silicon synapse unit costs *N_v_* + 1 clocks because of 2 stages in it. All of them finish their calculation at the $\left(\frac{N_{f}\times N_{v}^{2}}{4}+6\right)th$ clock. Thus the relationship between the period of an update step Δ*t* and the running clock of the system *f_s_* is described in the following Eq. (12).

(12) $$\Delta t=\frac{1}{f_{s}}\left(\frac{N_{f}\times N_{v}^{2}}{4}+6\right).$$

### 2.4 FPGA implementation

**Figure 7:**
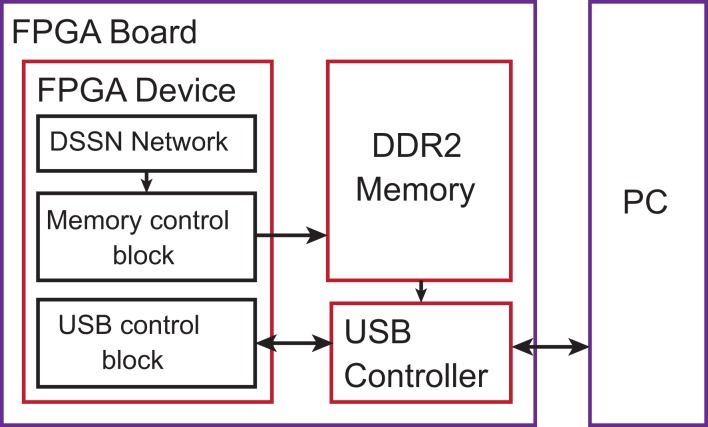
**The architecture of the whole system**.

We implemented our silicon neuronal network on a FPGA. The Digilent Atlys FPGA board equipped with Xilinx Spartan-6 LX45 FPGA is selected to construct an all-to-all connected 256-neuron network (*N_f_* = 16 and *N_v_* = 16). Here, we use block RAMs to store synaptic weights and 4 multipliers in parallel for the accumulator unit. The multipliers and a part of adders are implemented in the DSP elements. Device utilization after synthesis by ISE design tool is listed in Table 3. We integrated a communication module that transfers data between the PC and the FPGA device via the USB port. Control signals are sent to the FPGA and neuronal firing information of the network are sent back to the PC. The architecture of the total system is illustrated in Figure 7. The DDR2 memory is utilized as a buffer to avoid the speed conflict between data generation in the network and data transfer through the USB bus. Our silicon neuronal network starts calculation when it receives the start signal and initial state stimulus from the PC.

**Table 3:** **Device utilization on FPGA device**.

Logic utilization	Utilization	Available
Slice registers	14,198 (26%)	54,576
LUTs	18,556 (68%)	27,288
Block RAMs	73 (63%)	116

## 3 Results

**Figure 8:**
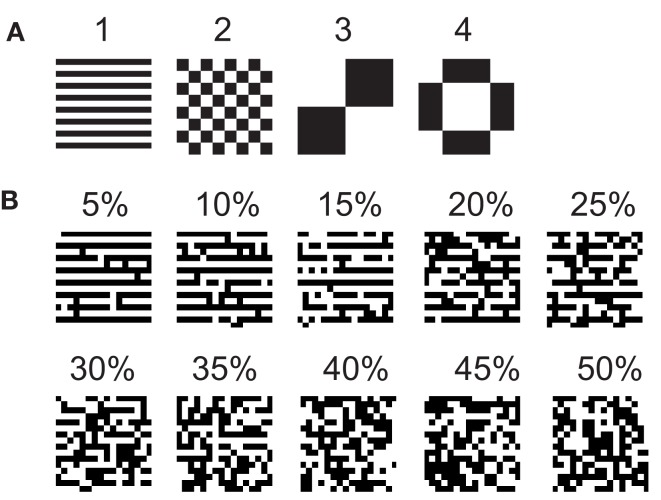
**(A)** Stored patterns and **(B)** a set of input patterns generated based on the stored pattern 1.

We evaluated the functionality of our silicon neuronal network circuit by constructing an auto-associative memory network, which retrieves stored memory patterns in response to an input similar to one of them. The auto-associative memory task is one of the most fundamental task for the fully connected silicon neuronal networks because the analysis of spike generation and phase locking are available to evaluate the properties of the network. This network is composed of 256 silicon neurons and 256^2^ synapses. We considered binary memory patterns denoted by $x_{i}^{u}\in \left\{-1,1\right\};$ $x_{i}^{u}$ represents the state of the *i*th neuron in the *u*th stored pattern. The weight matrix of this network is calculated by correlation learning using these patterns as follows (Hopfield, 1984).

(13) $$W_{ij}=\left\{\;\begin{array}{cc} \frac{1}{p}\overset{p}{\underset{u=1}{\sum }}x_{i}^{u}x_{j}^{u} & \text{when}\;i\neq j,\\ 0 & \text{when}\;i=j, \end{array}\right.$$

where *p* ≡ 4 is the number of the stored patterns. Those patterns we used here are shown in Figure 8A as black-white pictures with 16 × 16 pixels, where black and white represent $x_{i}^{u}=1$ and $x_{i}^{u}=-1,$ respectively. The external inputs we applied to the network are based on a stored pattern, but with certain amount of errors. We prepared input patterns with different amount of errors for each of 4 stored patterns by randomly inverting pixels. The error rates vary from 5 to 50% with 5% steps. We show an example of these patterns, which were generated from the stored pattern 1 in Figure 8B.

The overlap defined in (Domany and Orland, 1987; Aoyagi, 1995) was calculated to quantify the similarity between the state of neurons and a stored pattern. The following equations describe the overlap *M_u_* between the state of neurons and the *u*th stored pattern.

(14) $$M_{u}\left(t\right)=\frac{1}{N}|\overset{N}{\underset{j=1}{\sum }}x_{j}^{u}\exp\left(i\phi_{j}\left(t\right)\right)|,$$

where *N* is the number of neurons and Φ*_j_*(*t*) is the phase value of the *j*th neuron given at time *t* by Eq. (15) (Rosenblum et al., 2001).

(15) $$\phi_{j}\left(t\right)=2\pi k+2\pi \frac{t-t_{j}^{k}}{t_{j}^{k+1}-t_{j}^{k}},\left(t_{j}^{k}\leq t<t_{j}^{k+1}\right)$$

where $t_{j}^{k}$ is the time when the membrane potential of the *j*th neuron grows over the threshold and the transmitter starts to be released in response to the *k*th spike. All neurons in our network fire regularly where phase Φ*_j_*(*t*) codes the state of the *j*th neuron. Thus, according to Eq. (14), we defined that the network successfully retrieves the stored pattern if the relevant overlap equals to 1.

Because the synchronization between neurons is important in such phase-coded network, we analyzed it by the phase synchronization index (PSI) which is proposed in (Rosenblum et al., 2001). It is calculated by Eq. (16) and takes a value in [0, 1]. The full synchrony is detected if the PSI equals to 1.

(16) $$PSI\left(t\right)=\frac{1}{N}|\overset{N}{\underset{j=1}{\sum }}\exp\left(i2\phi_{j}\left(t\right)\right)|,$$

where Φ*_j_*(*t*) is the phase value defined by the Eq. (15). Here we chose the coefficient 2 in the exponential part to scale neuronal phases because phase differences between neurons are 0 or π when the input pattern coincides to one of the stored patterns.

We applied an impulse stimulus input *I*_stim_ for 16.875 ms which refracts an input pattern. The network is expected running 45 update steps in this period and one update step costs 0.375 ms when the clock is 2746.67 KHz according to the Eq. (12). In a neuron the initial *I*_stim_ corresponds to the pixel with value of 1 and −1, *I*_stim_ is large and small, respectively. These *I*_stim_ values are 0.125 and 0 for Class I neurons and 0.0425 and 0 for Class II. Then *I*_stim_ equals to 0.074 and 0.0295 for Class I and II neurons after the impulse. In this task, these *I*_stim_ were added to *I*_0_ for simplicity just after the system reset signal (clears registers for *v* and *n* to zero) is disactivated. In applications such as connection with event-based biomorphics sensors including silicon retina and cochlea (Liu and Delbruck, 2010), the stimulus input may be applied via the silicon synapses dedicated to receive external inputs and pulse width limiter circuits in case the sensors output too long pulse.

**Figure 9:**
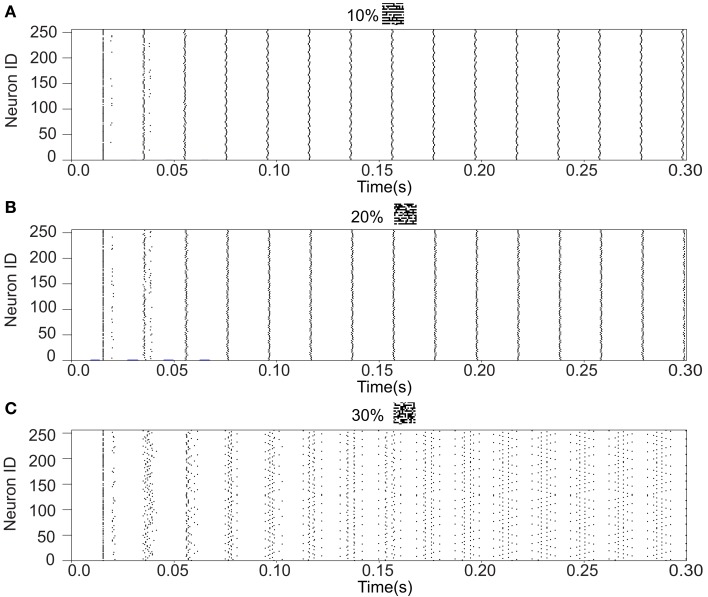
**Raster plots of the memory retrieval in the Class I mode and the input pattern includes 10, 20, and 30% errors shown in Figure 8B**. The input pattern is observed at 15.375 ms in both **(A–C)**. The stored pattern 1 and it’s reversed pattern alternately appear from 55.875 ms in **(A)**, and from 56.625 ms in (**B)**. However, they do not appear in **(C)**.

**Figure 10:**
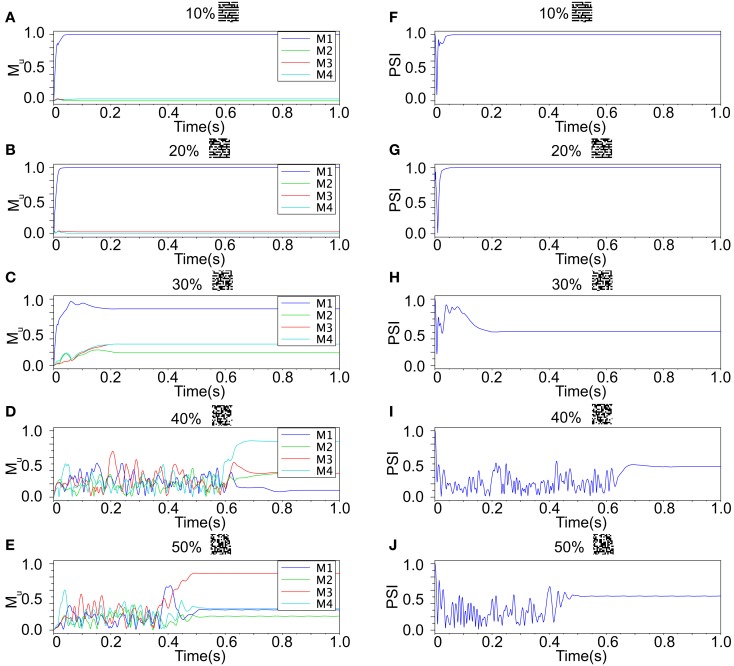
**Responses of associative memory on the Class I mode network when the patterns in Figure 8B was applied**. Planes **(A–E)** show the overlaps when the input pattern contains from 10 to 50% errors. In each plane, four colored curves labeled as M1, M2, M3, and M4 are overlaps between the state of the neurons and the stored pattern 1, 2, 3, and 4, respectively. Planes **(F–J)** show the synchronized properties when the input pattern contains from 10 to 50% errors.

**Figure 11:**
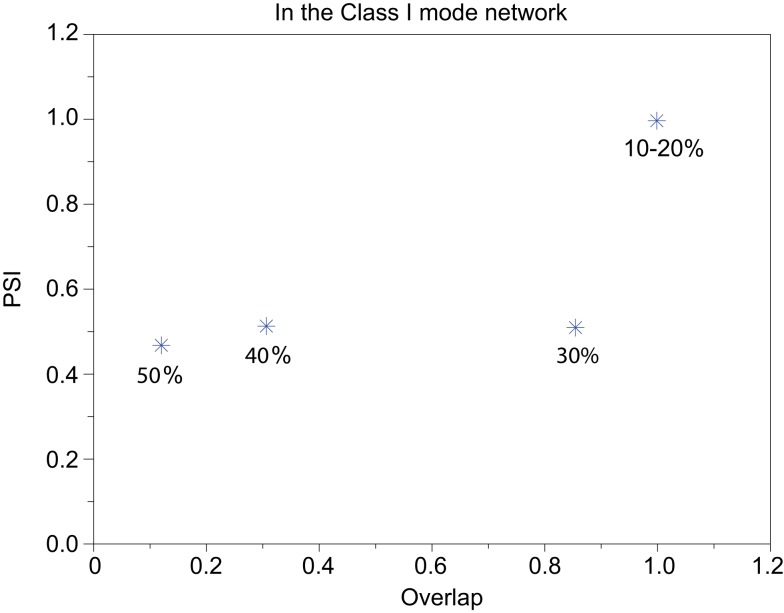
**Relevance of the overlap and the synchronization in Figure 10**.

We refer to our neuronal network as in the Class I mode when all of the neurons are in the Class I mode and the same to the Class II mode. Figure 9 shows the raster plots of the memory retrieval in the Class I mode and the input pattern includes Figure 9A 10%, Figure 9B 20%, and Figure 9C 30% errors (see Figure 8B). The input pattern appearing in the network at 15.375 ms. Then the neurons’ activity is controlled by the dynamics that depend on the value of the weights stored in the network. The stored pattern 1 in Figure 8A and it’s reversed pattern alternately appear from 55.875 ms in Figure 9A, from 56.625 ms in Figure 9B, and they do not appear in Figure 9C, which means unsuccessful memory retrieval. Figure 10 shows the process of memory retrieval and the properties of synchrony of the neural activities when the network is in the Class I mode and 5 of patterns in Figure 8B were applied as inputs. If the input pattern contains errors less than or equal to 20%, the network immediately achieves successful memory retrieval (*M*_1_ = 1) and maintains the retrieved pattern for the remaining time. If overlaps stabilize, the state of the network is assumed to be a steady state. In this state of the network, the stored pattern is exactly retrieved on each firing cycle (Figures 10A,B), and the PSI reaches unity, which indicates that the neural activities are fully synchronous(Figures 10F,G). When the input pattern contains 30% errors (Figure 10C), the overlap *M*_1_ transiently increases to unity at the beginning and then decreases to *M*_1_ ≈ 0.8538 around 0.19275 s, and the PSI also transiently increases to unity and then decreases according to the changes in the overlap (Figure 10H). When the errors are further increased (Figures 10D,E), an accurate stored pattern cannot be retrieved with errors remaining on the output pattern and the PSI is also largely decreased (Figures 10I,J). In Figure 11, we plotted the relation between the overlap and PSI, where PSI is 1 if our network retrieves a stored pattern completely.

**Figure 12:**
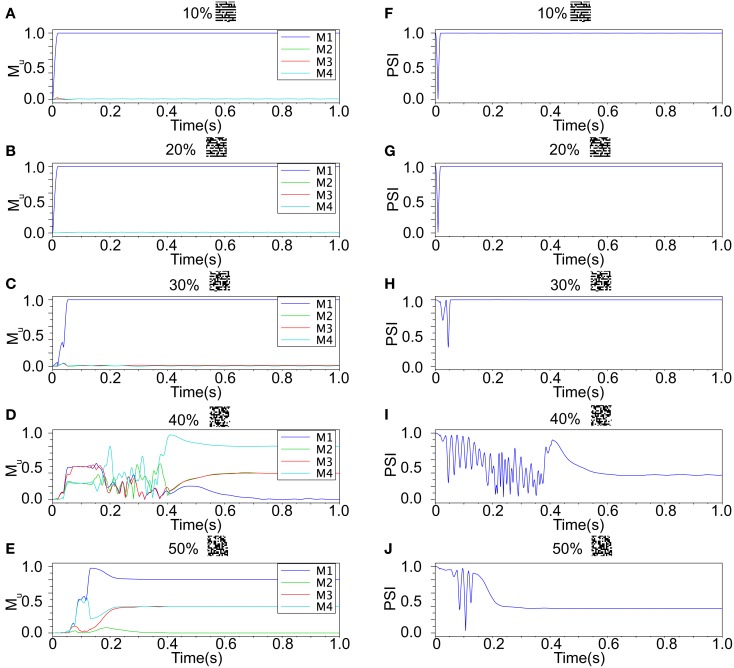
**Responses of associative memory on the Class II mode network when the patterns in Figure 8B was applied**. Planes **(A–E)** show the overlaps when the input pattern contains from 10 to 50% errors. In each plane, four colored curves labeled as M1, M2, M3, and M4 are overlaps between the state of the neurons and the stored pattern 1, 2, 3, and 4, respectively. Planes **(F–J)** show the synchronized properties when the input pattern contains from 10 to 50% errors.

**Figure 13:**
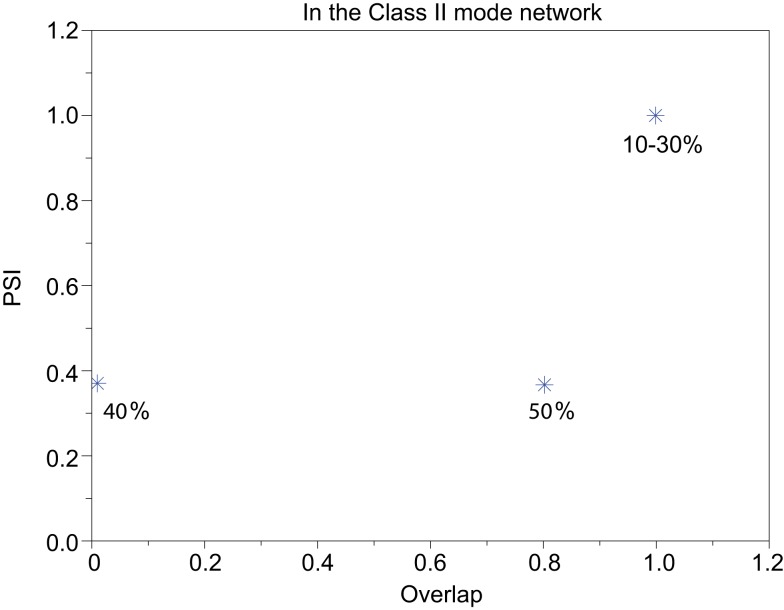
**Relevance of the overlap and the synchronization in Figure 12**.

Our neuronal network in the Class II mode achieves successful memory retrieval when the errors are less than or equal to 30% (Figures 12A–C) where the neurons exhibit synchronous activity (Figures 12F–H). When the errors in the input pattern are larger than 30%, the network cannot achieve successful memory retrieval (*M*_1_ < 1; Figures 12D,E), and the synchronization largely decreases to *PSI* ≈ 0.4 (Figures 12I,J). Thus the relationship between the performance of the memory retrieval and the synchrony in the Class II mode is similar to that in the Class I mode (Figure 13).

**Figure 14:**
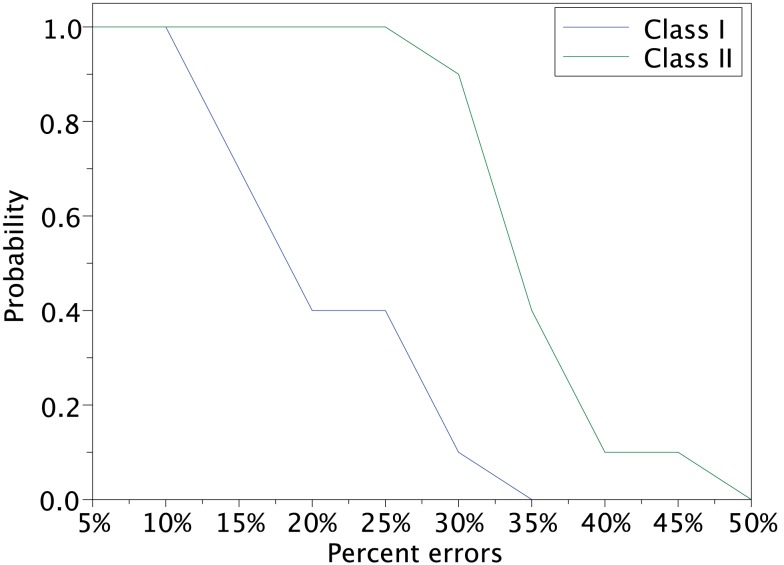
**Retrieving probability for the Class I and II mode networks**. Randomly generated 12 sets of patterns based of the 4 stored patterns (Figure 8A) were used as the input.

In order to investigate the reproducibility of the memory retrieval performance, we tested more cases to evaluate robustness against input errors in the Class I and II mode networks. We performed 12 trials of above experiment using 12 sets of input patterns which are generated based on 4 stored patterns and 3 sets for each stored pattern. The 10 different error rates varying from 5 to 50% of input patters are tested. We calculated the fraction of successful memory retrieval, which attains *M_u_* = 1 in the steady state of the network dynamics (Figure 14). In the Class I mode, our neuronal network achieves the successful memory retrieval in all the cases when errors in a input pattern are less than or equal to 10%; the fraction of successful memory retrieval decreases with errors increasing. The fraction becomes 0 when errors are larger than 40% in the Class I mode network. On the other hand, the Class II mode network shows completely successful memory retrieval even with the 25% errors. The fraction of the successful memory retrieval decreases but still keeps around 90% with the 30% errors, while it is only around 10% for the Class I mode network. These results suggest that the Class II mode network performs better than the Class I mode network when the associative memory task is executed.

## 4 Conclusion

We have reported our silicon neuronal network based on the digital operational circuit, which can be efficiently implemented in a FPGA device. Our silicon neuron is implemented by using the DSSN model where neuronal behaviors are abstracted using mathematical techniques so that it is capable of realizing behaviors in both Class I and II neurons with small number of multipliers to reduce the hardware resource requirement. Because the state variables are not reset in the spiking dynamics, it is expected that this model can describe the dependence of spike waveform on the stimulus far more effectively than the LIF and the IZH models where resetting of the variables is one of the points that reduce the complexity in their models and their implementations. It restricts dynamics in the spikes by assuming their maximum values are uniform. The IZH model actually can realize various neuronal activities including that of Class II neurons, which the LIF model cannot, though its spikes have a very similar waveform (Figure 3C). Meanwhile, simplicity in our model is maintained by reducing the number of multiplications, which is effective to realize compact digital circuit implementations. By utilizing the techniques of the phase plane and the bifurcation analyses, we successfully found the parameters for Class I and II models where the coefficients are selected in powers of two or sums of two such numbers. It allows us to replace the multiplications in our model by shift and add operations except for calculation of the square of variable *v*. Thus, our model can be implemented with single multiplier, which is the same to in (Cassidy and Andreou, 2008). Their circuit cannot realize the graded response of Class II neurons because they are implementing the IZH model. In addition, our silicon neuron circuit can be expanded to a 3-variable version with no additional multiplier, which can produce autonomous bursting similar to in the IZH model (Kobayashi et al., 2011). It was also shown that our model can reproduce very complex neuronal behaviors including chaotic ones by the fixed point representation (Kohno and Aihara, 2007) that requires less hardware resource than the floating point representation, which are used in (Thomas and Luk, 2009). Our silicon synapse model qualitatively describes process of the transmitter release, receptor activation, and generation of synaptic current described in the kinetic models (Destexhe et al., 1998). It was implemented without using multipliers by selecting Δ*t*α and Δ*t*β in power of two numbers. The salient feature of the synaptic output in our model is the time course of rise and decay that is dependent on the spike width. However it is usually neglected in other silicon neuronal networks (Cassidy and Andreou, 2008; Thomas and Luk, 2009; Arthur et al., 2012).

We constructed a fully connected network of 256 neurons on a Digilent Atlys FPGA board equipped with a Xilinx Spartan-6 LX45 FPGA. Calculating one step of a neuron needs 257 multiplications and 267 additions. This large amount of calculation was solved by the pipelined and parallel structure based on the tradeoff between hardware resource requirement and updating speed of the network.

The functionality of our silicon neuronal network and the significance of the Class II model in our silicon neuron were demonstrated by an auto-associative memory task. Its performance was evaluated by storing 4 patterns and applying inputs similar to them but including errors. The result shows that our silicon neuronal network has potential of retrieving the stored pattern even when the input pattern contains error and the neurons fire synchronously in case of successful retrieving. The Class II mode network has higher retrieving probability than the Class I mode network which is caused by differences of dynamical properties in Class I and II neurons. We can expect that the retrieving probability of our network is better than (Arthur et al., 2012) because it can only realize Class I neurons. It is known that one of the major difference between Class I and II neurons is the dependence of the spike form on the input strength. In Class II neurons, it depends strongly on the input, whereas it is almost constant in Class I neurons. We expect that this difference is playing at least a partial role in the performance of the auto-associative task, which will be elucidated in our future works. In this paper, our silicon neuronal network was tested only in its fully connected network topology without any adaptive learning rules. Because any connecting topology can be realized by disabling appropriate connections in the fully connected one, our system is ready to be tested in any settings. Under such restrictions, our results support that our silicon neuronal network can execute one of the most fundamental tasks for the neuronal networks and its distinctive feature of realizing Class II neurons can improve retrieving performance.

The custom neuromorphic chips investigated by both FACETs and NeuroGrid are the compact analog circuits which are composed of the silicon neurons based on the detailed neuron models (Bruderle et al., 2010; Choudhary et al., 2012). Their circuit is compact and consumes lower power but is generally sensitive to the noise and the fabrication mismatch. SpiNNaker simulates the detailed neuronal model which runs in the software on the embedded ARM processors with a high speed clock (Jin et al., 2010). Such systems generally consume higher power in comparison to our silicon neuronal network which is based on the optimized for implementation model and implemented by the compact and dedicated hardware running with a low speed clock. In SyNAPSE, the digital circuit implementation of the LIF model is used (Arthur et al., 2012), which we already have mentioned above. For real-time operation as an artificial nerve system, our silicon neuronal network requires very low clock frequency around several thousands of kilo hertzs. Each silicon neuron has 256 synaptic inputs, which will be increased up to about 10,000, which is a typical number of synaptic connection of a neuronal cell in the neocortex. In such case, the clock frequency will be about a hundred mega hertz (about 40 times faster than current frequency). Digital circuits with such range of clock frequency can be implemented by the near-threshold logic technology which consumes very low power. And it also consumes less power in cheap FPGAs. Thus our system can be suitably applied to robot controllers and compact intelligent sensor devices. For example, there is a possibility that our silicon neuronal network is connected to the event-based biomimetic sensors via additional silicon synapses dedicated to external inputs and realize an intelligent sensor such as retina-like image sensors. On the other hand, our silicon neuronal network can operate much more faster than the nerve system even in entry-level FPGA devices. Actually, our system can operate with 100 MHz system clock, which 40 times accelerated in comparison to the real-time operation. Thus, our system can be applied as a high speed simulator of neuronal networks composed of the qualitative neuron models, which is utilized as an important tool for the connectionists. Compared with the event-based network (Chicca et al., 2004), our network is expected to catch sensitive event information for its high speed operation and low power consumption.

In our future works, we will evaluate performance of our silicon neuronal network in the auto-associative task more in detail from theoretical viewpoints. It includes the comparison of the performance between our DSSN model’s and the IZH model’s networks as well as the evaluation of the memory capacity and the effect of introducing the STDP learning rules into both of the networks. This will elucidate more clearly how the neuron classes affect the performance of the auto-associative memory task. The large-scale network will also be pursued that can be implemented in a single FPGA chip, which will be applied to realizing intelligent sensors including retina-like image sensors. We expect possibility that the selectivity of neuron classes in our silicon neuronal network can improve such devices.

## Conflict of Interest Statement

The authors declare that the research was conducted in the absence of any commercial or financial relationships that could be construed as a potential conflict of interest.
